# Ocular injury awareness, knowledge, and safety practices among dental professionals, students, and supporting staff: A cross-sectional analysis

**DOI:** 10.1371/journal.pone.0350450

**Published:** 2026-07-14

**Authors:** Venisha Borkar, Ajinkya M. Pawar, Krishna Prasad Shetty, Alexander Maniangat Luke, Mohammad Fareed, Mohmed Isaqali Karobari

**Affiliations:** 1 Department of Conservative Dentistry and Endodontics, Nair Hospital Dental College, Mumbai, Maharashtra, India; 2 Department of Clinical Science, College of Dentistry, Ajman University, Al-Jruf, Ajman, United Arab Emirates; 3 Centre of Medical and Bio-Allied Health Science Research, Ajman University, Al-Jruf, Ajman, United Arab Emirates; 4 Department of Basic Medical Sciences, College of Medicine, AlMaarefa University, Diriyah, Riyadh, Saudi Arabia; 5 Research Center, Deanship of Scientific Research and Postgraduate Studies, Almaarefa University, Diriyah, Riyadh, Saudi Arabia; 6 Department of Conservative Dentistry and Endodontics, Saveetha Dental College and Hospitals, Saveetha Institute of Medical and Technical Sciences, Saveetha University, Chennai, Tamil Nadu, India; 7 Department of Conservative Dentistry and Endodontics, Faculty of Dentistry, University of Puthisastra, Khan Daun Penh, Phnom Penh, Cambodia; International Medical University, MALAYSIA

## Abstract

**Background:**

Ocular health is important in dentistry because the profession relies on visual acuity and has a high risk of splatter, aerosols, and projectiles. There is a great deal of promotion with respect to personal protective equipment (PPE), but little is known about how the awareness is turned into protective behaviour in dental practice in Indian contexts.

**Objective:**

To assess the extent of knowledge, awareness, and clinical practices related to ocular safety among dental professionals, students, and auxiliary staff in India, and to identify significant discrepancies between their theoretical knowledge and practical implementation.

**Methodology:**

An online cross-sectional survey was conducted among 512 dental professionals across various institutions in India by using a 27-item questionnaire that was diligently piloted and validated. The questionnaire serves to assess self-reported awareness, the incidence of ocular injuries, and patterns of eye protection for both clinicians and their patients.

**Results:**

Although 86.1% of respondents reported being familiar with eye safety regulations, 62.5% reported eye injuries during clinical procedures, mainly as a result of spatter, aerosol, and removing restorations, which doesn’t suggest lack of protection but lack of protective eyewear. Responsive to the conditions mentioned, only 42.6% were compliant overall and consistent in wearing protective eyewear, while 93.6% report acknowledging the need for protective eyewear. Additionally, 13.3% had observed ocular injuries in patients, and 38.35% admitted to not providing eye protection during procedures.

**Conclusion:**

There is a significant differential between the high awareness and poor uptake of ocular safety practices in Indian dentistry. This requires enhancing policy in institutions, introducing ocular protection in dental education and establishing standard procedures. Reducing preventable injuries and ensuring the safety of patients and practitioners is justified.

## Introduction

In the context of dental care, vision is our dominant sense accounting for about 75% of all sensory information we receive [1]. The all the visual sensory information we process in whatever task we are engaged with in dentistry, whether it be detecting caries, performing endodontic procedures, or completing surgery, good vision is essential to success [2,3]. Dental professionals also face various physical hazards including aerosols, splatter, flying objects, sharp instruments, and optical radiation that increase the potential for ocular injury [4]. The potential for ocular injury included acute injuries to the eye whereby bloodborne pathogens (Hepatitis B, Hepatitis C, and HIV) could easily be transmitted from client to dental professional [5,6]. Regular exposures to chemicals such as sodium hypochlorite, phosphoric acid, methyl methacrylate and radiation from curing lights and laser emitters also increase ocular injury risk [7–9].

To deal with this risk, supranational best practice recommendations from the WHO, ADA and OSHA for use include always wearing impact resistant protective eyewear with side shields or full-face shields, during every dental procedure [10]. Despite these recommendations, ocular injuries have been documented among dental professionals in various countries, including Greece [11], Nigeria [12], the United Kingdom [13], and Saudi Arabia [14,15], with frequencies of 38% to 73% and ongoing reports of ocular injuries still occurring. The ongoing report of ocular injuries, and the large frequency of ocular injuries in these studies, especially in high-income countries where recommendations are established, suggest a large gap between knowledge and practice.

In India, the disparity may be even greater due to systemic, educational, and infrastructural barriers. The Dental Council of India (DCI) does not require ocular safety training as either an undergraduate component or postgraduate training outcome, nor does the DCI impose any national policy requiring dental institutions to follow protective equipment protocols. Therefore, the implementation of safety practices varies from institution to institution, and region to region. A recent survey by Uppal et al. [16] in the National Capital Region showed that while 77% of dental professionals reported ocular protection was important, only 23% reported using protective eyewear consistently. Likewise, Arvind et al. [17] demonstrated that, although knowledge about ocular infections among Indian dentists was of moderate quality, actual practice of eye protection was poor.

Further research in other Indian states supports these findings. A cross-sectional survey of 150 dental practitioners in Bengaluru identified that just over half of respondents indicated wearing protective eyewear, while others did not wear any protection—this indicates a significant knowledge–practice gap [18]. In Maharashtra, a recent survey of dental students identified that only 15.5% could identify that it was essential to use eye protection for aerosol-generating procedures—still indicating a knowledge--practice gap in safety [19]. On a national scale, Nimma et al. [20] conducted an audit of eye safety protocols among dentists in India and found that 69% of respondents wore eye safety protection consistently, 21.6% wore it some of the time, and 9.6% indicated they did not wear any eye protection. These are clear systemic gaps in institutional enforcement; more importantly, even for practitioners who are aware of ocular hazards, there are major behavioural gaps.

Educational gaps may don’t help the situation. Malandkar et al. [21] investigated the knowledge, attitudes and practices associated with PPE in a cross-sectional study of 390 dental practitioners from India. Out of their findings it was 85% thought their work efficiency was affected by PPE use, and 89% stated diagnosed communication problems which illustrates strong barriers practically and educationally to be able to work safely.

Given a multitude of contributory factors—including poor training, lack of mandates and variation in access to equipment—there is an evident need to conduct an extensive national survey of ocular safety practices across the field of Indian dentistry. While mini regional studies exist, no large multicenter studies have been conducted to date which document self-reported awareness, injury events and compliance behaviour of the entire professional continuum—from faculty to students to supporting staff.

This investigation is, therefore, intended to explore knowledge, awareness and clinical practice regarding ocular safety measures across dental professionals and clinical career support staff in the entire Indian context. This study considers the breadth of knowledge–practice gaps across regions and professional roles in order to underpin targeted educational reform, institutional staff training plans and to provide the basis for a national safety-related policy to help lessen the incidence of avoidable occupational ocular injuries and incidents experienced in dentistry.

## Materials and methods

### Study design and ethical approval

The study was reported in accordance with the Strengthening the Reporting of Observational Studies in Epidemiology (STROBE) guidelines for cross-sectional studies. All dental professionals, students, and auxiliary personnel at one or more dental institutions and centers in India were invited to complete the questionnaire in awareness of this developmental research study. The study was a cross-sectional questionnaire-based survey of January 10 to July 10, 2024. Ethical approval was obtained from the Institutional Ethics Committee of Nair Hospital Dental College, Mumbai (Approval No: EC-209/CONS/ND112/2023; Date: 04/01/2024) and informed written consent was obtained from all participants with assurances of confidentiality.

### Questionnaire development and validation

A 27-item questionnaire was developed, drawing on knowledge from studies conducted in Saudi Arabia [15], Greece [11], and Nigeria [12]. These studies were selected as a foundation for the questionnaire due to their relevance and significance in the field of dentistry, providing insights into the prevalence of ocular injuries, eye protection practices, and occupational hazards specific to dental professionals in these regions. After contextually modifying the questionnaire for the Indian context, the instrument was assessed for content validity by a panel of dental experts and a statistician. A pilot study (N = 30 dental professionals) was then conducted to assess the clarity and fidelity of the survey. The final tool, which was assessed for internal reliability using Cronbach’s alpha generated a reliability of 0.81, suggesting good levels of internal consistency.

The questionnaire is called the Borkar and Pawar’s Questionnaire for Ocular Injuries and is copyright registered with the Copyright Office of India (ROC No.: L-131921/2023, registered on 11 Aug 2023). The questionnaire contains three parts:

Part I (5 questions) focused on demographic details, gathering information on participants’ demographic data, including gender, age, location/state, years of practice, and qualifications. The qualifications included options such as Faculty, Dental Practitioner, Dental Assistant, Dental Hygienist, and Dental Technician.

Part II (16 questions) assessed awareness of ocular safety among dental professionals and auxiliary workers. This section evaluated participants’ awareness of eye safety protocols, the importance of eye safety, and knowledge of potential causes of ocular injuries. Participants were queried about their experiences with ocular injuries, including frequency, causes, and first aid treatments employed. Additionally, participants were asked about their awareness of various eye safety equipment, their practices regarding the type and frequency of eye protection used, visits to an ophthalmologist, and any eye surgeries undergone.

Part III (6 questions) examined awareness of ocular safety concerning patients. This section focused on participants’ awareness of eye safety protocols for patients during procedures, the importance of eye safety, and their practices for patient eye protection.

### Linguistic and accessibility

Since the medium of instruction for dental education at institutions in India is English, the questionnaire was distributed in English without requiring translation. Nevertheless, all participants were asked to provide comment on the language clarity of the questionnaire during the pilot testing, and although not a substantial number of changes, minor changes were made after consideration.

### Sampling and recruitment

An online snowball sampling method was used for this study. The participants were contacted on professional dental forums, institutional mailing lists, and academic groups via WhatsApp, Telegram, and Instagram. The researchers put the information on a Google Form, where survey participants were asked to send the survey link to other colleagues and colleagues. The researchers used private messages as well as public and targeted posts for recruitment. To minimize the bias that may be inherent in snowball sampling, the initial distribution of the survey targeted a blend of urban and rural institutions of both public and private nature. The research team monitored completion of surveys intermittently, so they would ensure a variety of states were represented; they did not send out recruitment reminders once sufficient saturation was reached by region.

The study was designed and conducted in accordance with established methodological standards for cross-sectional survey research. The questionnaire demonstrated good internal consistency (Cronbach’s alpha = 0.81) after a multi-step process was conducted to establish its validity through both content validation by experts and pilot/testing.

An a priori sample size calculation was conducted to determine the proper number of subjects necessary to allow for appropriate statistical analyses prior to collecting data, using OpenEpi software based on known prevalence data in order to ensure that sufficient statistical power would be available for conducting analyses. Participants were recruited using a snowball convenience sampling method; however, several measures were taken to enhance the representativeness of the sample by recruiting dentists working in various geographic locations, various institutional types and roles within the dental professions in India.

### Sample size estimation

To establish the appropriate sample size, a specific formula was applied: Sample size n = [DEFF*Np(1-p)]/ [(d2/Z21-α/2*(N-1)+p*(1-p)]. The study’s parameters included a population size (N) of 1,000,000, an estimated frequency of the outcome factor in the population (p) of 65% ± 5%, and confidence limits set at 5% of 100 (d). The design effect (DEFF) was determined to be 1, as it was not relevant to this study. With a 95% confidence level, the calculated sample size was 512.

A 20% response rate was considered to account for potential non-responses. Consequently, the projected number of non-respondents was added to the original sample size to adjust the total sample size. Thus, the final sample size, obtained using the convenience sampling technique, was 614 (512 + 102). These results were derived using OpenEpi, Version 3, an open-source calculator—SSPropor. The reference for sample size estimation was obtained from by Uppal et al., [16].

### Statistical analysis

Data used in the study were analyzed using SPSS version 25.0 (IBM Corp., Armonk, NY, USA). Descriptive statistics were reported as frequencies and percentages, means and standard deviations. Normality was assessed using the Shapiro–Wilk test. Descriptive statistics for age and years of practice were compared by gender with independent t-tests, with awareness scores compared by professional role using one-way ANOVA. Chi-square (χ²) tests were used to assess associations between categorical variables, and alternatively Fisher’s exact test was used when expected cells were <5. Post hoc pairwise comparisons with Bonferroni correction were conducted when appropriate. Statistical significance was set at p < .05.

## Results

### Demographic details

When demographic factors were considered and compared across genders, no statistically significant differences were found among average age (males: 31.6 ± 10.2 years; females: 30.4 ± 9.6 years; t = 1.55, df = 510, p = 0.12) or years of professional experience (males: 7.8 ± 9.2 years; females: 6.9 ± 8.6 years; t = 1.70, df = 510, p = 0.09). On the contrary, since their professional role distribution was significantly different across males and females (χ² = 34.7, df = 4, p < 0.001), males were more likely to be practitioners (33.3%) or technicians (24.9%) and females were more likely to be hygienists (13.7%) or assistants (12.5%). The distribution of faculty members was more equal, with 37.3% of them being women and 39.5% being men. Notwithstanding similarities in age and experience, this data shows a clear gender disparity in dental occupations (Table 1).

**Table 1:** Demographic details and professional characteristics of the study participants (n = 512).

Variable	n (%) or mean ± SD		Statistical Test		p-value
**Gender – Female**	335 (65.4%)		Chi-square (χ²) test of independence		0.12
**Gender – Male**	177 (34.6%)		Chi-square (χ²) test of independence		0.12
**Mean age (years)**	30.8 ± 9.8		Independent t-test		0.12
**Years of practice**	7.2 ± 8.8		Independent t-test		0.09
**Dental Faculty**	195 (38.1%)		Chi-square (χ²) test of independence		0.32
**Dental Practitioners**	127 (24.8%)		Chi-square (χ²) test of independence		0.41
**Dental Technicians**	76 (14.8%)		Chi-square (χ²) test of independence		0.28
**Dental Assistants**	58 (11.3%)		Chi-square (χ²) test of independence		0.37
**Dental Hygienists**	56 (10.9%)		Chi-square (χ²) test of independence		0.29
**Variable**	**Male (n = 177)**	**Female (n = 335)**	**Statistical Test**	**Value (df)**	**p-value**
**Mean age (years)**	31.6 ± 10.2	30.4 ± 9.6	Independent t-test	t = 1.55 (df = 510)	0.12
**Years of practice**	7.8 ± 9.2	6.9 ± 8.6	Independent t-test	t = 1.70 (df = 510)	0.09
**Professional role**			χ² test of independence	χ² = 34.7 (df = 4)	<0.001*
Faculty	70 (39.5%)	125 (37.3%)			
Practitioners	59 (33.3%)	68 (20.3%)			
Technicians	44 (24.9%)	32 (9.6%)			
Assistants	16 (9.0%)	42 (12.5%)			
Hygienists	10 (5.6%)	46 (13.7%)			

### Gender distribution in categories of qualification

To gain a better understanding of demographic variation the gender distribution of occupational roles was assessed. The distribution of gender across six categories is presented in Table 2. Although females represented most of the total survey respondents, the ratio in which they represented each of the different types of qualification varied. For example, females appeared most often in their numbers as dental hygienists and assistants, while dental practitioners presented as being more evenly distributed between gender. Since descriptive attributes of occupational representation weren’t considered statistically significant, no statistical tests were performed.

**Table 2:** Gender-wise distribution of respondents based on qualification.

Qualification	Male n (%)	Female n (%)	Total n (%)
Dental Faculty	70 (35.9%)	125 (64.1%)	195 (38.1%)
Dental Practitioners	59 (46.5%)	68 (53.5%)	127 (24.8%)
Dental Technicians	44 (57.9%)	32 (42.1%)	76 (14.8%)
Dental Assistants	16 (27.6%)	42 (72.4%)	58 (11.3%)
Dental Hygienists	10 (17.9%)	46 (82.1%)	56 (10.9%)
Total	199 (38.9%)	313 (61.1%)	512 (100%)

### Geographical distribution of participants

**Fig 1:**
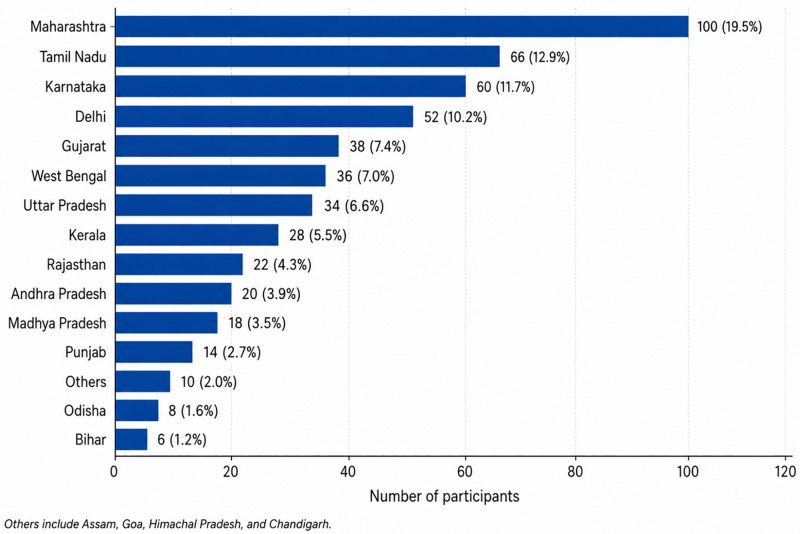
State-wise bar chart illustrating the geographic distribution of survey participants by state.

The survey included responses from dental professionals and auxiliaries from various states of India, indicating a wide geographic dispersion. As shown in Fig 1, the state-wise distribution of respondents demonstrated broad geographic participation across India. The highest number of responses was received from Maharashtra (100; 19.5%), followed by Tamil Nadu (66; 12.9%), Karnataka (60; 11.7%), and Delhi (52; 10.2%). Other states contributing responses included Gujarat (38; 7.4%), West Bengal (36; 7.0%), Uttar Pradesh (34; 6.6%), and Kerala (28; 5.5%). Fewer responses were received from Rajasthan (22; 4.3%), Andhra Pradesh (20; 3.9%), Madhya Pradesh (18; 3.5%), Punjab (14; 2.7%), Odisha (8; 1.6%), and Bihar (6; 1.2%). In addition, 10 responses (2.0%) were received from other states and Union Territories, including Assam, Goa, Himachal Pradesh, and Chandigarh. The broad geographical administration of the survey highlights the national relevance and the generalizability of results to the dental community across India. No significant differences were observed in gender distribution between high- and low-response states (p = 0.21).

### Awareness and importance of ocular safety guidelines pertaining to dental professionals

**Fig 2:**
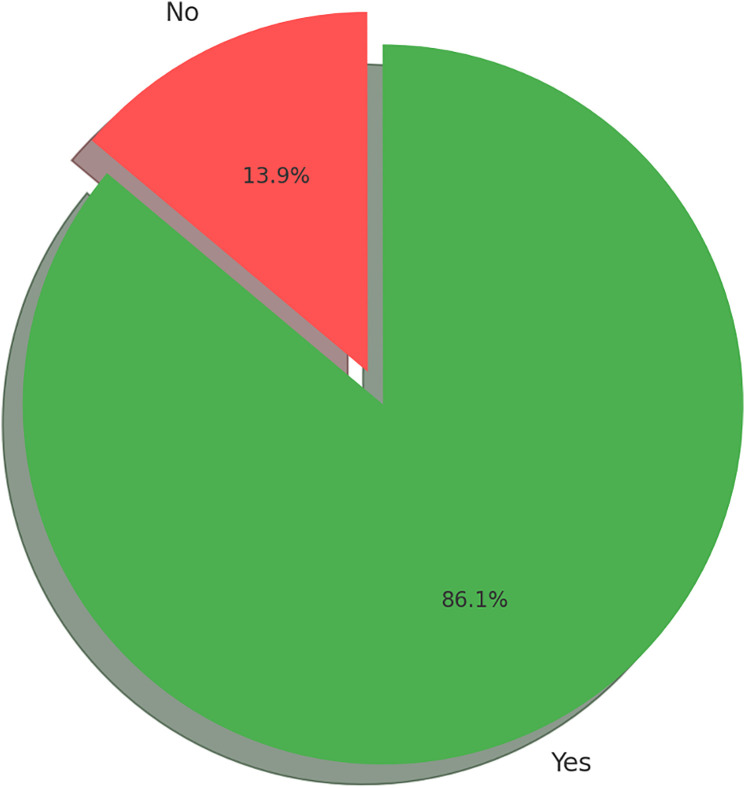
Participants’ awareness and perception of the importance of eye safety guidelines.

The analysis of eye safety awareness among the 512 participants revealed that 441 individuals (86.1%) affirmed their awareness of eye safety protocols when working with patients. Conversely, 13.9% of participants were not aware of such protocols (Fig 2). Notably, all 512 participants (100%) acknowledged the importance of eye safety protocols. A mean score of 9.1 ± 1.4 was observed when asked to rate the importance of ocular safety on a scale from 1 to 10, with 10 being the highest importance, which was fairly consistent value across participants with no significant difference by gender (p = 0.28) or role (ANOVA, p = 0.33).

A gender analysis of ocular injuries indicated a significantly larger percentage of males (69.5%) reported ocular insults compared to females (58.8%). This difference was borderline for significance (χ² = 3.41, p = 0.065). Regarding the frequency of injuries, males more often reported recurrent injuries, particularly at monthly or weekly frequencies, while females categorized their injury as having occurred “rarely” (χ² = 8.92, p = 0.012). The cause of reported incidents also varied by gender (χ² = 11.47, p = 0.042), with males most frequently attributing incidents to foreign bodies and laboratory procedures, while females most often attributed incidents to splatter or aerosols and removal of old restorations. Generally, first aid practices were similar across genders and irrigating with water immediately was the most frequently selected option (76.1% overall), although males were somewhat more likely to seek further care compared to females and this was not statistically significant (χ² = 2.37, p = 0.124). These results suggest modest but notable gender differences in the incidence, frequency, and etiological profile of ocular injuries among dental professionals; thus, it is important to all consider targeted injury prevention strategies (Table 3).

**Table 3:** Experience, frequency, causes, and first aid responses to ocular insults among participants.

Category	Response	Male n (%)	Female n (%)	Total n (%)	p-value (Chi-square)
Incidence	Experienced ocular injury	123 (69.5%)	197 (58.8%)	320 (62.5%)	0.065
	No injury	54 (30.5%)	138 (41.2%)	192 (37.5%)	
Frequency	Weekly	15 (8.5%)	5 (1.5%)	20 (3.9%)	<0.05
	Monthly	30 (16.9%)	44 (13.1%)	74 (14.5%)	
	Rarely	65 (36.7%)	188 (56.1%)	253 (49.4%)	
	Never	54 (30.5%)	83 (24.8%)	137 (26.8%)	
Cause	Splatter/aerosol	35 (19.8%)	64 (19.1%)	99 (19.3%)	0.42
	Foreign bodies	54 (30.5%)	33 (9.9%)	87 (17.0%)	
	Old restoration removal	28 (15.8%)	49 (14.6%)	77 (15.1%)	
	Lab procedures	38 (21.5%)	18 (5.4%)	56 (11.0%)	
	Other causes	22 (12.4%)	66 (19.7%)	88 (17.2%)	
First aid	Wash with water	95 (53.7%)	295 (88.1%)	390 (76.1%)	<0.001
	Cold compress	8 (4.5%)	14 (4.2%)	22 (4.3%)	
	Blink eye	10 (5.6%)	15 (4.5%)	25 (4.9%)	
	Medical help	40 (22.6%)	35 (10.4%)	75 (14.7%)	

### First-aid and medical response

In answer to questions about what first-aid response to take with an injury to the eyes, most (76.1%) chose immediate irrigation with water, 14.7% asked for medical attention, and the rest chose blinking or cold compresses as an initial response.

### Eye protection practices of practitioners

The use of protective eyewear varied considerably: 48.8% opted for safety glasses, 33.8% wore prescription eyeglasses, and 14.4% reported using visors, while 3.1% indicated that they did not use any ocular protection. While awareness was high (93.6%) only 42.6% reported habitual use, which was often dependent upon the procedure performed. Gender did not emerge as a statistically significant predictor (p = 0.18) of habitual use; however the auxiliary, was statistically less likely to report habitual use than faculty (χ² = 12.9, p = 0.005).

### Ophthalmologic consultations and surgical history

Analysis of ophthalmologic care patterns indicated there were no meaningful differences between males and females in terms of visit frequency, reasons for contact and surgical history. Males reported seeing an ophthalmologist monthly (12.4%) compared to females (6.9%) but the majority of each group reported visiting infrequently (74.6% males, 77.9% females (χ² = 2.14, p = 0.343)). With respect to the purpose of visits both males and females exhibited comparable patterns, with 24.3% of males and 20.6% of females reported visiting only because of an injury, and having an exam or check-up was slightly more common for females (43.9 vs. 45.8%; χ² = 1.92, p = 0.289). Surgical history was also comparable with 11.9% male and 9.7% female reported a previous minor or major ocular surgical procedure (χ² = 0.48, p = 0.487). Overall, it appears that the patterns of utilization of ophthalmologic care were similar for males and females without meaningful differences (Table 4).

**Table 4:** Ophthalmologist visits, reasons for visits, and history of eye surgeries among participants.

Question	Response	Male n (%)	Female n (%)	Total n (%)	χ² (p-value)
How frequently do you visit an ophthalmologist?	Monthly	22 (12.4%)	23 (6.9%)	45 (8.8%)	χ² = 2.14 (p = 0.343)
	Rarely	132 (74.6%)	261 (77.9%)	393 (76.8%)	
	Never	23 (13.0%)	51 (15.2%)	74 (14.5%)	
Do you visit an ophthalmologist only after an ocular injury or also go for a regular checkup?	Ocular injury	43 (24.3%)	69 (20.6%)	112 (21.9%)	χ² = 1.92 (p = 0.289)
	Check-up	81 (45.8%)	146 (43.9%)	227 (44.3%)	
	Both	34 (19.2%)	56 (16.8%)	90 (17.6%)	
	Not applicable	19 (10.7%)	64 (19.2%)	83 (16.2%)	
Have you undergone any minor or major eye surgeries?	Yes	21 (11.9%)	33 (9.7%)	54 (10.5%)	χ² = 0.48 (p = 0.487)
	No	155 (88.1%)	303 (90.3%)	458 (89.5%)	

### Response consistency and data integrity

A cross check of responses indicated a difference in answers regarding the incidence of ocular injuries. A total of 192 participants reported no ocular injuries in the past year; however, only 137 chose “never” in the injury frequency selection, and 35 picked “not applicable”. This difference in responses may be somewhat due to answering questions differently, and/or interpreting questions differently, which would be a limitation to the self-administered questionnaire format.

### Awareness of ocular safety pertaining to patients

**Fig 3:**
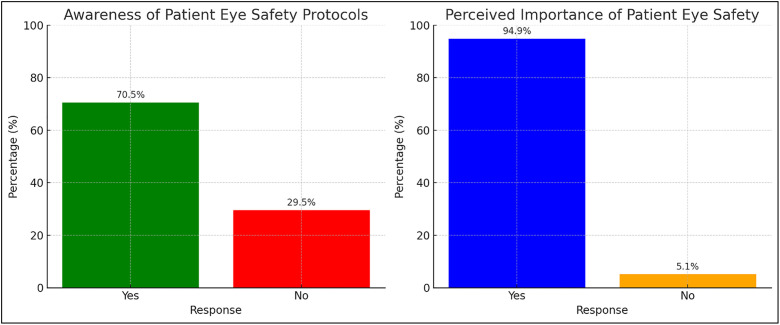
Awareness and perceived importance of patient eye safety protocols.

The results showed 70.5% of participants reported awareness of eye safety practices for patients, and there was a statistically significant difference in participant awareness levels (faculty = 81.6794 and auxiliaries = 72.7272) (χ² = 10.2, p = 0.006). Almost all participants (94.9%) felt that patient eye safety was an important concept, with a mean rating of 8.35 ± 1.98 (Fig 3).

Statistically significant differences were found in the provision of type of patient eye protection (χ², p < 0.001). Safety glasses were the most common patient eye protection offered (58.0%) and were offered more than spectacles, or visors (post-hoc, p < 0.001). As such, very few participants offered the patient with prescription spectacles (1.9%, p = 0.04), or visors (1.7%, p = 0.08). In addition, an alarming 38.4% of participants stated they did not provide patients with any eye protection when conducting dental procedures, which also was statistically significant (p = 0.001). These findings suggest an overwhelming reliance on safety glasses whilst highlighting a major risk to patient safety and share the concern for patient safety with a considerable amount of patients clearly without any patient eye protection (Table 5).

**Table 5:** Patient ocular injuries, provided eye protection, and frequency of eye safety glasses use.

Question	Response	Frequency (n)	Percent (%)	Statistical Test	Value (df)	p-value
Have the patients faced any ocular injury while you were working on them?	Yes	68	13.3	χ² test	χ² = 9.6 (df = 1)	0.002
	No	444	86.7			
What type of eye protection do you provide for your patients?	Prescribed spectacles	10	1.9	χ² test (overall)	χ² = 134.2 (df = 3)	<0.001
	Visors	9	1.7			
	Safety glasses	297	58.0			
	None	196	38.4			
Frequency of use of eye safety glasses or shields	Never	115	22.5	χ² test	χ² = 16.8 (df = 4)	0.002
	Rarely	104	20.3			
	Occasionally	114	22.3			
	Sometimes	106	20.7			
	Always	73	14.3			

## Discussion

The study presents a striking contradiction: more than four out of five participants stated they knew about ocular safety practices, yet less than half stated they routinely wore eye protection. Knowledge-practice gaps are common, but our study demonstrates that they are deeply engrained in India. The lifetime incidence of 62.5% ocular injuries was observed, which is greater than reported from Pakistan (60%) [22] and some areas of Saudi Arabia (38%) [23], but less than was reported from Riyadh (86.8%) [15], Greece (73%) [11] and Nigeria (72.3%) [12]. Almost half of participants with injuries reported “rare” injuries, while 14.5% reported injuries on a monthly basis. These findings provide evidence that even occasional non-compliance in wearing protective eyewear has real clinical implications. The major causes of ocular injuries were scaling and polishing, cavity-preparation, and laboratory procedures. These findings reinforce previous findings that high-speed rotors and laboratory procedures produce debris and aerosols that can lead to ocular insults [24]. Additionally, retinal damage from curing lights indicates that injury risks not only included projectile injuries but also phototoxic exposures [25].

The contradiction is in why there is such a high level of awareness of risk but a lack of consistent protective behavior. Our finding is similar to that of Malandkar et al. [21] who report, 85% of dentists feel PPE impairs their efficiency. When asked what those barriers were, PPE discomfort, fogging and communication were notable. Similar barriers were reported by hygienists in the United States [26]. In other words, these ergonomic and psychosocial factors contribute to a perceived obstacle that protection interferes with clinical performance. In situations like this it seems a workshop and continuing education to reframe safety as a facilitator of professional efficiency could be worthwhile.

The findings of this study regarding ocular safety compliance and awareness fall in line with overall trends seen throughout other healthcare fields and professions. Just as dental healthcare practitioners can be at risk for injuries due to high-velocity projectiles as well as aerosol exposure that is contaminated, there are multiple studies that show that other Healthcare Workers (HCWs), also experience serious risk of injury through occupation hazards while not having a high level of compliance with safety standards [27,28]. A paradoxical aspect of infection control is that although dental providers and dental students consistently show greater compliance than nursing or medical staff, their perception of overall occupational risk is often lower than their medical or nursing colleagues [27].

Generally, nurses in all medical fields express high awareness when dealing with biological dangers. However, because they occur so often at the bedside, nurses will have a greater chance of having a percutaneous injury from handling these biological dangers [29]. However, ocular exposure through infectious splash effects is still a common threat to workers in all health care fields. Data suggests that a substantial portion of physicians and nurses underutilize ocular protection, frequently attributing this to physical discomfort or a perceived lack of necessity during non-invasive procedures [27,28]. This indicates that the safety gaps identified among dental professionals in the current study are emblematic of a systemic healthcare challenge, where theoretical awareness does not always manifest as consistent protective behavior [28,30].

These issues are exacerbated by systemic challenges. It is important to note that the present study did not directly assess or stratify institutional safety policies across participating sites. However, given the wide geographic distribution of respondents and inclusion of multiple institutional types, it is likely that variability in local safety protocols, enforcement practices, and availability of protective equipment existed. This heterogeneity may have influenced individual compliance behaviors and could partly explain the observed inconsistencies in ocular safety practices. Unlike infection control, ocular protection is not required by the Dental Council of India nor woven into training curricula as a core competency. The lack of systematic policy leads to commoditized institutional levels of variability and individualization of ocular safety. In contrast, compliance in contexts of stated policy exceeds to 60% [14], significantly above Indian rates. Without federal audits or accreditation requirements, eyewear protection is optional and unsafe behaviors continue.

Cultural and behavioral systems can provide more explanations. Dentistry in India has hierarchical systems: senior practitioners do not wear eyewear and students and aides follow suit. Arvind et al. [17] identified that clinicians may acquire knowledge but do not model safe practice to their patients, and this leads to a continual cycle of non-adherence. Youthful dentists may also underestimate risk associated with their own behaviors — the “invulnerability bias” — in models of Greek [11] and Nigerian [12] dentists. People also consider appearance in their decision-making process: most individuals will purposely not use bulky eyewear as they feel it diminishes their professionalism, consistent with behavioral studies illustrating that aesthetic and matters of social concern will take precedence over rational and systematic safety behaviors [31].

In addition to personal factors, practical obstacles also need to be addressed. The expense for quality visors or prescription glasses that ensure safety for work practice, is high especially for small independent practices. Practitioners in Bengaluru consistently cited as barriers, estimated to be just over 40% of practitioners indicating that price is a barrier [18]. While financial concerns also play a role, there are also misconceptions. Many dentists feel safe with regular spectacles, however, the work by Farrier et al. [32] has shown that rates of injury among spectacle users are higher than those wearing actual safety glasses. These misconceptions create barriers requiring educational and other initiatives to not only raise awareness, but to correct false beliefs and showing a cost-benefit of appropriate prevention.

Encouragingly, 93.6% of participants were aware of face shields and safety glasses and 42.6% indicated consistent use of either — higher than survey results from Nigeria (7.4%) [12], Saudi Arabia (24.5%) [23], or the National Capital Region of India (23%) [16]. However, compliance remains lower than other areas of the world with stronger institutional restrictions. For example, compliance is greater than 60% for regular face shield and safety glasses use in parts of Saudi Arabia [14]. Most often used were safety glasses (48.8%) and we again saw similar NCR survey results (47%) [16]. Visors remained rare (14.3%), and a few surveyed acknowledged they used no eye protection at all. Overall, these results reaffirm that compliance is dependent on organizational policy, cultural perceptions, and barriers to access.

Patient protection is arguably the most concerning finding. While 94.9% recognized the significance of patient protection, almost 40% of respondents indicated they had never provided any ocular protection and 13.3% had witnessed ocular injuries to patients. This reflects international trends: Oleksiak et al. [33] found that while 96% of dental students knew how ocular transmission occurs, fewer than 1 in 5 dental students routinely provided ocular protection. Our findings confirm that in the absence of a systemic level of enforcement, patient safety is left to the discretion of the clinician – not a viable approach.

These results together point to the need for multi-tiered interventions. On the educational level, workshops, simulated training, and integrating ocular protection into the curriculum would help normalize ocular protection as a feature of professionalism. On the institutional level, mandatory policies, annual audits, and the provision of affordability and standardization of the eyewear could enforce compliance. On the regulatory level, the Dental Council of India including ocular protection as part of its inspection and accreditation on the training institutions and private clinics would support this. Structural support, along with a cultural change, is needed to help raise awareness into ongoing safe practice.

When interpreting the findings of this study, several limitations should be acknowledged. First, due to the cross-sectional design of the study, no cause-and-effect relationships can be established. Second, self-reported data can result in self-report bias (i.e., participants may choose to report socially desirable responses). Third, participants’ recollections of their ocular injuries/clinical practices may also present a problem with recall bias and affect the accuracy of their reported frequencies and behaviours. Finally, since a snowball sampling technique was used, caution should be taken when generalizing the results beyond this sample to the overall dental community.

However, this research project has some strengths. The size of the sample, the geographic representation of the sample, and the agreement among respondents across groups increase the confidence in these results. Thus, the conclusions drawn from this study are based on the study data (e.g., the sizeable difference between awareness and compliance and the large incidence of ocular injuries) and indicate that targeted, systemic, and educational interventions should be made for dentists in the future.

## Conclusion

This research indicates that there is a continued gap between knowledge and compliance regarding ocular safety protocol among many Indian dentists, students, auxiliaries and patients, thus both, dentists and patients are exposed to harm that could be prevented. Future research needs to go beyond cross-sectional surveys to longitudinal and interventional designs testing the impact of educational programs and workshops (based on simulation) that are ergonomic driven as well as cost- subsidized personal protective equipment. Comparative studies across geographical regions and within institution will be useful in describing systemic barriers, such as cost, habits of the culture and any form of institutional leadership in hindering compliance.

Ocular safety must be part of policy at the various levels of the curriculum and an integrated competency for both undergraduate and postgraduate education. This education must also be supported by enforceable national standards, routine audits, and accreditation. More crucially, along with supporting affordability initiatives, institutional leaders should also consider investing in ocular safety training to make it a requirement annually. By taking a multi-faceted approach to address both behavioural and systemic barriers through education, policy, and institutional action, dentistry in India has the ability to combat the risk of ocular injuries and develop sustainable behaviour around ocular safety for both providers and patients.

## Supporting information

S1 File Data set. (XLSX)

S2 File Questionnaire. (DOCX)

S3 File STROBE checklist. (DOCX)
