# Supplementary material for: Ocular injury awareness, knowledge, and safety practices among dental professionals, students, and supporting staff: A cross-sectional analysis
Source: PLoS One. 2026 Jul 14;21(7):e0350450. doi: 10.1371/journal.pone.0350450 (PMC13367719; doi:10.1371/journal.pone.0350450)
Supplement: S2 File — (DOCX) [file pone.0350450.s002.docx]

**Questionnaire**

By filling this form participants giving the consent to use the data for study (participants assured of confidentiality)

1. **Demographic details**

Gender:

Age:

Location/State:

Years of Practice:

Year of practice/ Qualification:

- II nd Year
- III rd Year
- IV th Year
- Intern
- Postgraduate
- Faculty
- Dental practitioner
- Dental Hygienist
- Dental Assistant
- Dental Technician

1. **Awareness of ocular safety pertaining to the dental professionals, students, and auxiliary staff**
2. Are you aware of eye safety protocols for yourself while working on a patient?

- Yes
- No

1. Do you think it is important ?

- Yes
- No

1. If yes, then rate the importance on a scale of 1-10.

_________

1. Which among the following do you think can lead to ocular insults in dentistry? (Can choose multiple options)

- Foreign Bodies
- Removal of old restorations
- Splatter or aerosol containing blood and saliva
- Lab procedures/ denture work
- Scaling and polishing
- Trauma from wires or burs
- Instrument cleaning
- Cavitation preparations
- Tooth extractions

1. Have you ever experienced ocular insults?

- Yes
- No

1. If yes, what is the frequency of these insults?

- Weekly
- Week and a half
- Monthly
- Month and a half
- Rarely
- Never

1. What procedures have caused these insults to you? (Can choose multiple options)

- Scaling and polishing
- Cavity preparations
- Tooth extractions
- Orthodontic treatment
- Lab procedures / denture work
- Instrument cleaning
- Instrument cleaning
- Removal of old restoration
- Not applicable

1. What do you do as a first aid treatment after an eye injury due to any of the above reasons?

- Immediately wash the eye with lots of water
- Apply cold compress in case of swellings
- Blink the eye repeatedly for removal of foreign particles
- Bandage the eye
- Get medical help
- Take medications for pain

1. Are you aware of various types of face shields and glasses used for eye protection

- Yes
- No

1. Frequency of use of eye safety glasses or shields.

- Never
- Rarely
- Occasionally
- Sometimes
- Always

1. What type of eye protection do you use?

- Visors
- Safety glasses
- Prescribed spectacles
- None

1. If you use prescribed spectacles, has the power of the lenses increased over the years?

- Yes
- No
- Not applicable

1. How frequently do you visit an ophthalmologist?

- Monthly
- Rarely
- Never

1. Do you visit an ophthalmologist only after an ocular injury or also go for a regular check up?

- Ocular injury
- Check up
- Both
- Not applicable

1. Have you undergone any minor or major eye surgeries?

- Yes
- No

1. If yes, what was the underlying cause for the surgery?

_____________________

1. **Awareness of ocular safety pertaining to the Patients.**
2. Are you aware of your patient’s eye safety protocols while working on them?

- Yes
- No

1. Do you think it is important ?

- Yes
- No

1. If yes, then rate the importance on a scale of 1-10.

_________

1. Have the patients faced any ocular injury while you working on them?

- Yes
- No

1. What type of eye protection do you provide for your patients

- Prescribed spectacles
- Visors
- Safety glasses
- None

1. Frequency of use of eye safety glasses or shields.

- Never
- Rarely
- Occasionally
- Sometimes
- Always
