# Supplementary material for: Ocular injury awareness, knowledge, and safety practices among dental professionals, students, and supporting staff: A cross-sectional analysis
Source: PLoS One. 2026 Jul 14;21(7):e0350450. doi: 10.1371/journal.pone.0350450 (PMC13367719; doi:10.1371/journal.pone.0350450)
Supplement: Attachment — Submitted filename: Comments.pdf [file pone.0350450.s006.pdf]

Abstract

The abstract is long and includes some repetition. For instance, details about specific percentages could be shortened without losing clarity.

Introduction

The rationale for conducting this study specifically in the Indian context is underdeveloped. Although the introduction cites several international studies, there is little discussion of the local situation in India. For instance, the manuscript does not sufficiently explore whether:

- There is a documented lack of adherence to eye safety protocols among Indian dental professionals;
- Dental institutions in India have uniform safety standards or mandates related to ocular protection;
- Prior Indian studies (if any exist) have shown gaps in awareness or behavior;
- Regional variations in practice or access to protective equipment may affect outcomes.

This omission weakens the justification for conducting a large-scale cross-sectional study in India.

Methodology

Provide a clearer explanation of how potential sampling biases were mitigated.

Result

The analysis remains descriptive throughout. There is no comparative statistical testing between groups (e.g., by gender or role).

Discussion

- The discussion is descriptive and could benefit from deeper critical reflection on “why” awareness doesn’t translate into safer practice.
- It lacks commentary on systemic issues such as policy enforcement, institutional protocols, or educational gaps.
- Sociocultural or behavioral explanations for poor compliance are missing.

- Critically explore barriers to implementation despite high awareness—e.g., discomfort, cost, peer influence, time constraints.
- Discuss the potential role of dental colleges and councils in mandating eye protection training

Conclusion

Does not outline specific future research directions or implications for policy.

Reference

Ensure uniform formatting per PLOS ONE
