# Supplementary material for: Ocular injury awareness, knowledge, and safety practices among dental professionals, students, and supporting staff: A cross-sectional analysis
Source: PLoS One. 2026 Jul 14;21(7):e0350450. doi: 10.1371/journal.pone.0350450 (PMC13367719; doi:10.1371/journal.pone.0350450)
Supplement: Attachment — Submitted filename: PLOS review.docx [file pone.0350450.s004.docx]

Interesting study

1. For the questionnaires which were drawn from Saudi Arabia, Greece and Nigeria was the questionnaire validated? If yes what was the Cronbachs alpha value obtained?

2. Was the questionnaire translated into any local language?

3. Kindly provide the complete questionnaire used here.

4. Kindly provide gender wise distribution for the respondents based on qualification to see if gender wise differences exist.

5. Exactly where was the study conducted is not clear, as the geographic variation may also need to be accounted.

6. Why no correlation statistics is performed?
