# Supplementary material for: Ocular injury awareness, knowledge, and safety practices among dental professionals, students, and supporting staff: A cross-sectional analysis
Source: PLoS One. 2026 Jul 14;21(7):e0350450. doi: 10.1371/journal.pone.0350450 (PMC13367719; doi:10.1371/journal.pone.0350450)
Supplement: Attachment — Submitted filename: Comments Addressed.docx [file pone.0350450.s007.docx]

**To**

**The Editor**

**Dear Prof,**

We sincerely appreciate the time and effort the editor and reviewers have dedicated to evaluating our manuscript. Their insightful comments and constructive suggestions have significantly enhanced the clarity, structure, and overall quality of our work. We are grateful for their valuable feedback, which has helped us refine our discussion and improve the organization of our review. Their expertise and thoughtful recommendations have strengthened our study, and we deeply appreciate their contribution to this research. Thank you for your support and guidance throughout the review process.

**ACADEMIC EDITOR:**

I thank you for submitting an interesting manuscript. However, there are a few major queries raised by the reviewers' which need to be addressed.

***Our Response.*** *We genuinely appreciate the Academic Editor for their valuable review and constructive comments. We have carefully addressed every comment and have revised the manuscript accordingly to improve clarity, coherence, and scientific rigor.*

**Reviewer #1: Interesting research.**

Abstract:

**Comment.** Well written and easy to understand. Just consider rewriting the first sentence for clarity. Suggested revision:

"Since approximately 75% of sensory data is processed visually, maintaining ocular health is critically important in the field of dentistry."

***Our Response.*** *We appreciate the suggestion. We concur that the modification enhances clarity, and the abstract's opening language has been amended appropriately.*

Introduction (Page 10, first and second paragraphs):

**Comment.** The paragraph starting with "On March 19, 2020, the World Health Organization..." and the one starting with "In February 2003, the British..." are both informative. However, I recommend presenting the information in chronological order for better flow and coherence.

***Our Response.*** *Thank you for this insightful observation. The said introduction section has been updated to lay out the content in chronological order. The 2003 sentence was removed to aid the flow of the section, and the paragraph has been rewritten to provide a historical review of the guidelines and standards, beginning with early recognition of the situation and culminating with the WHO's COVID-19 recommendations published in 2020.*

Materials and Methods:

**Comment.** Clear and well-structured.

***Our Response.*** *We appreciate your positive comments.*

Results:

**Comment.** Clear, well written, with good use of graphs and tables.

***Our Response.*** *We appreciate your positive remarks on the Results section.*

Discussion:

**Comment.** Consider clarifying the following sentence:
"The survey identified that 1.8% of participants never employed eye protection, while 18.8% used it sporadically, depending on the specific procedure."
You might ask: Could this result be influenced by differences in institutional policies, cultural factors, or national safety standards?

***Our Response.*** Thank you for highlighting this important point. We have revised the sentence to not only clarify the reported figures but also include potential contextual factors that may influence such behaviour. The updated version now reads: *“The survey found that 1.8% of participants never used eye protection, 18.8% reported only using eye protection inconsistently and depending on the procedure. These results may be indicative of variability in institutional policies, cultural attitudes toward safety, or national standards and regulations for protective equipment in various regions.”*

Conclusion:

**Comment.** Well written.

***Our Response.*** *Thank you. We appreciate your positive feedback and have retained the conclusion as is.*

**Reviewer #2**

**Comment.** Thank you very much for this research. It is an important topic. Nevertheless, there is a great need to rewrite this paper in a proper way. I have highlighted the points that needed to be fixed as the following. These points do not exclude your hard effort but rather aim to improve the work.

***Our Response.*** *We appreciate your kind thoughts about the significance of our study and your recognition of the work that went into it. We sincerely value your insightful and helpful criticism. Your thorough recommendations have been very helpful to us in enhancing the manuscript's general coherence, organization, and scientific calibre. Every point you raised has been thoroughly considered, and the manuscript has been updated as a result. Below are our answers to the specific remarks you made.*

Abstract:
**Comment.** Shorten the introduction section and explicitly state the aim of the study in the final sentence of the introduction. In the methodology section, clarify that the questionnaire was derived from a previous study. Do not mention the countries of the original study, as this may mislead the reader into thinking that data were collected from those countries. In the methodology, briefly indicate the country of the participants and whether the data collection was conducted in a single center or across multiple sites. In the methodology, the reported participant number (614) is unclear—does this refer to invitations or actual participants? Please clarify and report the final number of participants along with the actual response rate. In the sentence "Healthcare workers demonstrated significant awareness of patient ocular safety (70.5%) and its importance (average rating 8.35 ± 1.98)": if no statistical test was used, replace the word "significant" with "relatively high", as "significant" implies a statistical comparison. If a test was conducted, the p-value should be provided.

***Our Response.*** *We appreciate the reviewer's wise recommendations. In light of this, the abstract has been revised.*

Introduction:
**Comment.**

- The introduction is too long and can be shortened in several parts, especially paragraphs 3 and 4.
- No previous studies assessing knowledge or awareness of eye injuries were mentioned. Including such references would strengthen the rationale.

***Our Response.*** *We truly value the insightful criticism provided by the reviewer. We hope that the updated introduction upholds the standards and enhances the manuscript's overall quality.*

Methodology:
**Comments.**

- The section does not specify the country of the target population or the data collection setting. Is the study nationwide, local, or international? Please clarify.
- Indicate whether the questionnaire was adopted directly from previous studies conducted in Saudi Arabia, Greece, and Nigeria, or whether it was only conceptually based on those studies.
- Was a pilot study conducted to test the questionnaire before data collection? If yes, please provide details.
- The sentence "In this study, the Borkar and Pawar’s Questionnaire..." is unclear. Is this an additional tool used? Or is it the same questionnaire developed earlier? Please clarify.
- There is no description of the answer format (e.g., yes/no, multiple choice, Likert scale). Please specify the type and range of responses.
- There is insufficient information on data recruitment. It appears that data were collected online via social media—but how were participants reached? Did the authors use personal accounts? Which platforms were used? Were participants invited through public posts or private messages? This part should be placed earlier in the methodology, not under sample size estimation.
- The phrase "a mix of convenience and snowball sampling methods" is inaccurate. It should be classified as snowball sampling.
  The writing can be made more concise and direct. Several sentences are redundant and can be simplified.

***Our Response.*** *We appreciate the reviewer's thorough and helpful criticism. We hope that these thorough changes adequately address all of the concerns that were pointed out.*

Results:

**Comments.**

- The sentence "The study comprised 512 participants, of whom 177 (34.6%) were male and 335 (65.4%) were female, with a p-value of <0.05 indicating a statistically significant difference in gender distribution within the sample" is incorrect. A p-value is not appropriate here since this is purely descriptive data with no independent/dependent variable comparison.
- Figure 1 is unnecessary; the information can be reported numerically in Table 1.
- Although the methods section states that location or state data were collected, the results do not include this information. Please ensure consistency.
- The section on awareness is poorly written. For example, the score “average importance rating of 9.0703 ± 1.39012” is unclear. Is this a scale from 1 to 10? What do higher and lower scores indicate? Also, reporting four decimal places is inappropriate; limit it to one or two decimals. Figure 3 is unnecessary.
- The detailed procedure description (e.g., listing all sources of ocular injury) belongs in the methodology, not in the results.
- The use of p-values in Table 2 is incorrect. If no statistical test was applied, p-values should be removed.
- There is a contradiction in responses to questions 5, 6, and 7 in Table 2. For example, question 5 states that 192 participants had no ocular injury, but in question 6, 137 reported never experiencing ocular injury, and in question 7, 35 selected "not applicable." This inconsistency suggests fluctuations in participant responses and should be addressed.
- Figures 4 and 5 are unnecessary, and the reported p-values are invalid, as no statistical tests appear to have been conducted.

***Our Response.*** *We appreciate the reviewer's thorough comments. We have taken note of every remark and believe that these adjustments fully address the reviewer's issues while enhancing the Results section's integrity and clarity.*

References:

**Comments.**

- Several references are outdated. As a general rule, avoid citing sources older than 2005 unless absolutely necessary (e.g., seminal works).
- Examples of outdated references:
- Reference 1 (1978) – 47 years old, Reference 5 (1997), Reference 12 (1993), Reference 16 (1945) – 80 years old.
- Please replace these with more recent and relevant literature.

***Our Response.*** *We appreciate*

Reviewer #3:

**Comment.** Interesting study

***Our Response.*** We sincerely thank the reviewer for the positive comment and valuable suggestions.

**Comment.** For the questionnaires which were drawn from Saudi Arabia, Greece and Nigeria was the questionnaire validated? If yes what was the Cronbachs alpha value obtained?

***Our Response.*** The questionnaire was contextually adapted for the Indian population and validated through a pilot study. The Cronbach’s alpha value obtained was **0.81**, indicating good internal consistency.

**Comment.** Was the questionnaire translated into any local language?

***Our Response.*** As the participants were dental professionals and support staff trained in English, **no translation was required**, and the questionnaire was administered in English.

**Comment.** Kindly provide the complete questionnaire used here.

***Our Response.*** The questionnaire is structured in three parts and is described in the manuscript. However, we will be happy to provide the full questionnaire as **Supplementary Material** as per journal requirements.

**Comment.** Kindly provide gender wise distribution for the respondents based on qualification to see if gender wise differences exist.

***Our Response.*** Gender-wise distribution across different qualifications has been provided in **Table 2**, as requested.

**Comment.** Exactly where was the study conducted is not clear, as the geographic variation may also need to be accounted.

***Our Response.*** The study was conducted across **multiple dental institutions in India**. Geographic distribution of respondents has been detailed in the Results section and visualized in **Figure 1**.

**Comment.** Why no correlation statistics is performed?

***Our Response.*** Thank you for this suggestion. As this study was exploratory and primarily descriptive in nature, correlation analysis was not initially planned. However, we acknowledge the value of such analysis and will consider including it in future extended studies.
