# Supplementary material for: Ocular injury awareness, knowledge, and safety practices among dental professionals, students, and supporting staff: A cross-sectional analysis
Source: PLoS One. 2026 Jul 14;21(7):e0350450. doi: 10.1371/journal.pone.0350450 (PMC13367719; doi:10.1371/journal.pone.0350450)
Supplement: Attachment — Submitted filename: Response to Comments.docx [file pone.0350450.s008.docx]

**Response to Comments**

We appreciate the Editor’s insightful and constructive comments, which have greatly enhanced our manuscript. Below, we offer a detailed response to each point, including references to specific sections of the revised manuscript where changes have been implemented.

**Abstract**

**Comment 1.**
The abstract is long and includes some repetition. Details about specific percentages could be shortened without losing clarity.

**Response:**
We have refined the abstract by eliminating redundant details and retaining only the essential percentages to emphasize key findings (e.g., awareness 86.1%, ocular injuries 62.5%, compliance 42.6%). Repetition has been removed, allowing the abstract to convey the core message more succinctly (see Abstract in the revised manuscript).

✅ **Addressed.**

**Introduction**

**Comment 2.**
The rationale for conducting this study in the Indian context is underdeveloped. There is little discussion of local adherence to protocols, institutional standards, or prior Indian studies.

**Response:**
We have revised the Introduction to strengthen the rationale within the Indian context. In particular, we highlight that the Dental Council of India does not mandate ocular safety training and cite several Indian studies (Uppal et al., Arvind et al., Nimma et al., along with regional surveys in Bengaluru and Maharashtra). These studies uncover systemic gaps, regional disparities, and insufficient adherence to protective practices across India (pages 3–5).

✅ **Addressed.**

**Methodology**

**Comment 3.**
Provide a clearer explanation of how potential sampling biases were mitigated.

**Response:**
We added explicit details on bias mitigation: initial survey distribution targeted both urban/rural and public/private institutions, state-level representation was monitored, and reminders were withheld once saturation was reached (see *Sampling and Recruitment*, page 8).

✅ **Addressed.**

**Results**

**Comment 4.**
The analysis is descriptive; no comparative testing between groups (e.g., gender, role).

**Response:**
We included comparative statistical analyses throughout the *Results*:

- Gender vs. role distribution (χ² = 34.7, p < 0.001, Table 1).
- Gender-based differences in ocular injury incidence, frequency, and causes (χ² tests with p-values, Table 3).
- Differences in patient safety practices across groups (χ² tests, Table 5).
  These strengthen the analytical depth and go beyond descriptive reporting (pages 9–15).

✅ **Addressed.**

**Discussion**

**Comment 5**
The discussion is descriptive and lacks deeper reflection on why awareness doesn’t translate into practice. It also omits systemic, sociocultural, and policy barriers.

**Response:**
We substantially revised the *Discussion* to provide critical insights:

- **Ergonomic and psychosocial barriers** (e.g., PPE discomfort, fogging, communication issues, as noted by Malandkar et al.).
- **Cultural influences** (hierarchical modeling, appearance concerns, invulnerability bias).
- **Systemic challenges** (lack of Dental Council of India mandates, variability across institutions).
- **Financial and misconception barriers** (cost of visors, false sense of safety from spectacles).
- **Policy implications** (role of institutional audits, enforceable standards, affordability initiatives).

These revisions provide a comprehensive explanation of the knowledge–practice gap and highlight multi-tiered solutions (pages 16–20).

✅ **Addressed.**

**Conclusion**

**Reviewer comment:**
The conclusion does not outline specific future research directions or policy implications.

**Response:**
We revised the *Conclusion* to explicitly recommend:

- Longitudinal and interventional research (e.g., educational workshops, simulation-based interventions).
- Comparative regional studies to identify systemic barriers.
- Integration of ocular safety into undergraduate and postgraduate curricula as a core competency.
- Development of enforceable national standards, affordability initiatives, and institutional audits (page 21).

✅ **Addressed.**

**References**

**Reviewer comment:**
Ensure uniform formatting per PLOS ONE.

**Response:**
All references have been reformatted to ensure consistency with journal style. Numbering has been checked and aligned sequentially.

✅ **Addressed.**

**Summary**

All reviewer concerns have been comprehensively addressed in the revised manuscript through:

- Concise and focused abstract,
- Strengthened Indian context and justification,
- Clearer methodology with bias control,
- Comparative statistical analyses,
- Critical and multi-level discussion of barriers,
- Specific research and policy recommendations in conclusion,
- Standardized references.

We believe these revisions have significantly improved the manuscript and aligned it with the reviewer’s expectations.
