# Supplementary material for: Ocular injury awareness, knowledge, and safety practices among dental professionals, students, and supporting staff: A cross-sectional analysis
Source: PLoS One. 2026 Jul 14;21(7):e0350450. doi: 10.1371/journal.pone.0350450 (PMC13367719; doi:10.1371/journal.pone.0350450)
Supplement: Attachment — Submitted filename: Response_to_comments_auresp_3.docx [file pone.0350450.s009.docx]

Dear Prof,

We appreciate the Editors and Reviewers' insightful and constructive comments, which have greatly enhanced our manuscript. Below, we offer a detailed response to each point, including references to specific sections of the revised manuscript where changes have been implemented.

**Reviewer 5 comments**

**Comment:** The manuscript must describe a technically sound piece of scientific research with data that supports the conclusions. Experiments must have been conducted rigorously, with appropriate controls, replication, and sample sizes. The conclusions must be drawn appropriately based on the data presented.

***Our Response: We thank the reviewer for this important comment. The manuscript has been revised to further clarify the study's methodological rigour. Specifically, we have detailed the questionnaire validation process, including pilot testing and internal consistency (Cronbach’s alpha = 0.81), and expanded the description of the a priori sample size calculation using OpenEpi to ensure adequate statistical power. We have also clarified the statistical approach, including the appropriate selection of tests based on data distribution and the use of post hoc corrections. Finally, the conclusions have been carefully aligned with the presented data, particularly the observed discrepancy between awareness, compliance, and incidence of ocular injuries, to ensure that all inferences are directly supported by the results.***

**Comment:** The authors may consider briefly expanding the limitations section to explicitly acknowledge potential self-reporting and recall bias.

***Our Response: We thank the reviewer for this valuable suggestion. The limitations section has been revised to explicitly acknowledge the potential for self-report bias, including socially desirable responses, as well as recall bias in participants’ reports of past ocular injuries and clinical practices. These additions improve the transparency and interpretability of the study findings.***

**Comment:** A short comparison with similar occupational safety studies in other healthcare professions could strengthen the discussion.

***Our Response: We thank the reviewer for this insightful suggestion. The Discussion section has been revised to include a brief comparison with occupational safety practices in other healthcare professions, particularly among physicians and nursing staff. This addition highlights that the observed knowledge–practice gap is consistent with broader trends in healthcare, thereby strengthening the contextual interpretation of our findings.***

**Comment:** Clarification on whether institutional safety policies varied across participating sites would add useful context.

***Our Response: We thank the reviewer for this important observation. The Discussion section has been revised to clarify that institutional safety policies were not directly assessed or stratified across participating sites. We have also acknowledged that variability in local protocols, enforcement, and resource availability likely exists and may have influenced the observed differences in compliance, thereby providing additional context to the findings.***
