# Supplementary material for: Ocular injury awareness, knowledge, and safety practices among dental professionals, students, and supporting staff: A cross-sectional analysis
Source: PLoS One. 2026 Jul 14;21(7):e0350450. doi: 10.1371/journal.pone.0350450 (PMC13367719; doi:10.1371/journal.pone.0350450)
Supplement: Peer review history

# Peer review: Decision Letter 0

**DOI:** 10.1371/journal.pone.0350450.r001

**Authors:** Tanay Chaubal

7 Jun 2025

PONE-D-25-16205

Ocular Injury Awareness, Knowledge, and Safety Practices Among Dental Professionals, Students, and supporting Staff: A Cross-Sectional Analysis

PLOS ONE

Dear Dr. Karobari,

Thank you for submitting your manuscript to PLOS ONE. After careful consideration, we feel that it has merit but does not fully meet PLOS ONE’s publication criteria as it currently stands. Therefore, we invite you to submit a revised version of the manuscript that addresses the points raised during the review process.

==============================

**ACADEMIC EDITOR:**  I thank you for submitting an interesting manuscript. However, there are a few major queries raised by the reviewers' which need to be addressed.

==============================

Please submit your revised manuscript by Jul 22 2025 11:59PM. If you will need more time than this to complete your revisions, please reply to this message or contact the journal office at plosone@plos.org. When you're ready to submit your revision, log on to https://www.editorialmanager.com/pone/ and select the 'Submissions Needing Revision' folder to locate your manuscript file.

Please include the following items when submitting your revised manuscript:

- A rebuttal letter that responds to each point raised by the academic editor and reviewer(s). You should upload this letter as a separate file labeled 'Response to Reviewers'.
- A marked-up copy of your manuscript that highlights changes made to the original version. You should upload this as a separate file labeled 'Revised Manuscript with Track Changes'.
- An unmarked version of your revised paper without tracked changes. You should upload this as a separate file labeled 'Manuscript'.

If you would like to make changes to your financial disclosure, please include your updated statement in your cover letter. Guidelines for resubmitting your figure files are available below the reviewer comments at the end of this letter.

If applicable, we recommend that you deposit your laboratory protocols in protocols.io to enhance the reproducibility of your results. Protocols.io assigns your protocol its own identifier (DOI) so that it can be cited independently in the future. For instructions see: https://journals.plos.org/plosone/s/submission-guidelines#loc-laboratory-protocols. Additionally, PLOS ONE offers an option for publishing peer-reviewed Lab Protocol articles, which describe protocols hosted on protocols.io. Read more information on sharing protocols at https://plos.org/protocols?utm_medium=editorial-email&utm_source=authorletters&utm_campaign=protocols.

We look forward to receiving your revised manuscript.

Kind regards,

Tanay Chaubal

Academic Editor

PLOS ONE

Journal Requirements:

When submitting your revision, we need you to address these additional requirements.

1. Please ensure that your manuscript meets PLOS ONE's style requirements, including those for file naming. The PLOS ONE style templates can be found at https://journals.plos.org/plosone/s/file?id=wjVg/PLOSOne_formatting_sample_main_body.pdf and https://journals.plos.org/plosone/s/file?id=ba62/PLOSOne_formatting_sample_title_authors_affiliations.pdf

2. Please include a complete copy of PLOS’ questionnaire on inclusivity in global research in your revised manuscript. Our policy for research in this area aims to improve transparency in the reporting of research performed outside of researchers’ own country or community. The policy applies to researchers who have travelled to a different country to conduct research, research with Indigenous populations or their lands, and research on cultural artefacts. The questionnaire can also be requested at the journal’s discretion for any other submissions, even if these conditions are not met.  Please find more information on the policy and a link to download a blank copy of the questionnaire here: https://journals.plos.org/plosone/s/best-practices-in-research-reporting. Please upload a completed version of your questionnaire as Supporting Information when you resubmit your manuscript.

3. Please provide additional details regarding participant consent. In the ethics statement in the Methods and online submission information, please ensure that you have specified what type you obtained (for instance, written or verbal, and if verbal, how it was documented and witnessed). If your study included minors, state whether you obtained consent from parents or guardians. If the need for consent was waived by the ethics committee, please include this information.

4. We note that you have indicated that there are restrictions to data sharing for this study. For studies involving human research participant data or other sensitive data, we encourage authors to share de-identified or anonymized data. However, when data cannot be publicly shared for ethical reasons, we allow authors to make their data sets available upon request. For information on unacceptable data access restrictions, please see http://journals.plos.org/plosone/s/data-availability#loc-unacceptable-data-access-restrictions.

Before we proceed with your manuscript, please address the following prompts:

a) If there are ethical or legal restrictions on sharing a de-identified data set, please explain them in detail (e.g., data contain potentially identifying or sensitive patient information, data are owned by a third-party organization, etc.) and who has imposed them (e.g., a Research Ethics Committee or Institutional Review Board, etc.). Please also provide contact information for a data access committee, ethics committee, or other institutional body to which data requests may be sent.

b) If there are no restrictions, please upload the minimal anonymized data set necessary to replicate your study findings to a stable, public repository and provide us with the relevant URLs, DOIs, or accession numbers. Please see http://www.bmj.com/content/340/bmj.c181.long for guidelines on how to de-identify and prepare clinical data for publication. For a list of recommended repositories, please see https://journals.plos.org/plosone/s/recommended-repositories. You also have the option of uploading the data as Supporting Information files, but we would recommend depositing data directly to a data repository if possible.

Please update your Data Availability statement in the submission form accordingly.

5. In the online submission form, you indicated that the datasets used and/or analysed during the current study available from the corresponding authors on reasonable request.

All PLOS journals now require all data underlying the findings described in their manuscript to be freely available to other researchers, either 1. In a public repository, 2. Within the manuscript itself, or 3. Uploaded as supplementary information.

This policy applies to all data except where public deposition would breach compliance with the protocol approved by your research ethics board. If your data cannot be made publicly available for ethical or legal reasons (e.g., public availability would compromise patient privacy), please explain your reasons on resubmission and your exemption request will be escalated for approval.

6. Please remove all personal information, ensure that the data shared are in accordance with participant consent, and re-upload a fully anonymized data set.

Note: spreadsheet columns with personal information must be removed and not hidden as all hidden columns will appear in the published file.

Additional guidance on preparing raw data for publication can be found in our Data Policy (https://journals.plos.org/plosone/s/data-availability#loc-human-research-participant-data-and-other-sensitive-data) and in the following article: http://www.bmj.com/content/340/bmj.c181.long.

[Note: HTML markup is below. Please do not edit.]

Reviewers' comments:

Reviewer's Responses to Questions

**Comments to the Author**

1. Is the manuscript technically sound, and do the data support the conclusions?

The manuscript must describe a technically sound piece of scientific research with data that supports the conclusions. Experiments must have been conducted rigorously, with appropriate controls, replication, and sample sizes. The conclusions must be drawn appropriately based on the data presented.

Reviewer #1: Yes

Reviewer #2: Partly

Reviewer #3: Yes

**********

2. Has the statistical analysis been performed appropriately and rigorously?

Reviewer #1: I Don't Know

Reviewer #2: No

Reviewer #3: Yes

**********

3. Have the authors made all data underlying the findings in their manuscript fully available?

The PLOS Data policy requires authors to make all data underlying the findings described in their manuscript fully available without restriction, with rare exception (please refer to the Data Availability Statement in the manuscript PDF file). The data should be provided as part of the manuscript or its supporting information, or deposited to a public repository. For example, in addition to summary statistics, the data points behind means, medians and variance measures should be available. If there are restrictions on publicly sharing data—e.g. participant privacy or use of data from a third party—those must be specified.

Reviewer #1: Yes

Reviewer #2: No

Reviewer #3: Yes

**********

4. Is the manuscript presented in an intelligible fashion and written in standard English?

PLOS ONE does not copyedit accepted manuscripts, so the language in submitted articles must be clear, correct, and unambiguous. Any typographical or grammatical errors should be corrected at revision, so please note any specific errors here.

Reviewer #1: Yes

Reviewer #2: Yes

Reviewer #3: Yes

**********

5. Review Comments to the Author

Please use the space provided to explain your answers to the questions above. You may also include additional comments for the author, including concerns about dual publication, research ethics, or publication ethics. (Please upload your review as an attachment if it exceeds 20,000 characters)

Reviewer #1: Interesting research.

Abstract:

Well written and easy to understand. Just consider rewriting the first sentence for clarity. Suggested revision:

"Since approximately 75% of sensory data is processed visually, maintaining ocular health is critically important in the field of dentistry."

Introduction (Page 10, first and second paragraphs):

The paragraph starting with "On March 19, 2020, the World Health Organization..." and the one starting with "In February 2003, the British..." are both informative. However, I recommend presenting the information in chronological order for better flow and coherence.

Materials and Methods:

Clear and well-structured.

Results:

Clear, well written, with good use of graphs and tables.

Discussion:

Consider clarifying the following sentence:

"The survey identified that 1.8% of participants never employed eye protection, while 18.8% used it sporadically, depending on the specific procedure."

You might ask: Could this result be influenced by differences in institutional policies, cultural factors, or national safety standards?

Conclusion:

Well written.

Reviewer #2: Thank you very much for this research. It is an important topic. Nevertheless, there is a great need to rewrite this paper in a proper way.

I have highlighted the points that needed to be fixed as the following.

These points do not exclude your hard effort but rather aim to improve the work.

Abstract:

Shorten the introduction section and explicitly state the aim of the study in the final sentence of the introduction.

In the methodology section, clarify that the questionnaire was derived from a previous study. Do not mention the countries of the original study, as this may mislead the reader into thinking that data were collected from those countries.

In the methodology, briefly indicate the country of the participants and whether the data collection was conducted in a single center or across multiple sites.

In the methodology, the reported participant number (614) is unclear—does this refer to invitations or actual participants? Please clarify and report the final number of participants along with the actual response rate.

In the sentence "Healthcare workers demonstrated significant awareness of patient ocular safety (70.5%) and its importance (average rating 8.35 ± 1.98)": if no statistical test was used, replace the word "significant" with "relatively high", as "significant" implies a statistical comparison. If a test was conducted, the p-value should be provided.

Introduction:

The introduction is too long and can be shortened in several parts, especially paragraphs 3 and 4.

No previous studies assessing knowledge or awareness of eye injuries were mentioned. Including such references would strengthen the rationale.

Methodology:

The section does not specify the country of the target population or the data collection setting. Is the study nationwide, local, or international? Please clarify.

Indicate whether the questionnaire was adopted directly from previous studies conducted in Saudi Arabia, Greece, and Nigeria, or whether it was only conceptually based on those studies.

Was a pilot study conducted to test the questionnaire before data collection? If yes, please provide details.

The sentence "In this study, the Borkar and Pawar’s Questionnaire..." is unclear. Is this an additional tool used? Or is it the same questionnaire developed earlier? Please clarify.

There is no description of the answer format (e.g., yes/no, multiple choice, Likert scale). Please specify the type and range of responses.

There is insufficient information on data recruitment. It appears that data were collected online via social media—but how were participants reached? Did the authors use personal accounts? Which platforms were used? Were participants invited through public posts or private messages? This part should be placed earlier in the methodology, not under sample size estimation.

The phrase "a mix of convenience and snowball sampling methods" is inaccurate. It should be classified as snowball sampling.

The writing can be made more concise and direct. Several sentences are redundant and can be simplified.

Results:

The sentence "The study comprised 512 participants, of whom 177 (34.6%) were male and 335 (65.4%) were female, with a p-value of <0.05 indicating a statistically significant difference in gender distribution within the sample" is incorrect. A p-value is not appropriate here since this is purely descriptive data with no independent/dependent variable comparison.

Figure 1 is unnecessary; the information can be reported numerically in Table 1.

Although the methods section states that location or state data were collected, the results do not include this information. Please ensure consistency.

The section on awareness is poorly written. For example, the score “average importance rating of 9.0703 ± 1.39012” is unclear. Is this a scale from 1 to 10? What do higher and lower scores indicate? Also, reporting four decimal places is inappropriate; limit it to one or two decimals. Figure 3 is unnecessary.

The detailed procedure description (e.g., listing all sources of ocular injury) belongs in the methodology, not in the results.

The use of p-values in Table 2 is incorrect. If no statistical test was applied, p-values should be removed.

There is a contradiction in responses to questions 5, 6, and 7 in Table 2. For example, question 5 states that 192 participants had no ocular injury, but in question 6, 137 reported never experiencing ocular injury, and in question 7, 35 selected "not applicable." This inconsistency suggests fluctuations in participant responses and should be addressed.

Figures 4 and 5 are unnecessary, and the reported p-values are invalid, as no statistical tests appear to have been conducted.

References:

Several references are outdated. As a general rule, avoid citing sources older than 2005 unless absolutely necessary (e.g., seminal works).

Examples of outdated references:

Reference 1 (1978) – 47 years old

Reference 5 (1997)

Reference 12 (1993)

Reference 16 (1945) – 80 years old

Please replace these with more recent and relevant literature.

Reviewer #3: Interesting study

1. For the questionnaires which were drawn from Saudi Arabia, Greece and Nigeria was the questionnaire validated? If yes what was the Cronbachs alpha value obtained?

2. Was the questionnaire translated into any local language?

3. Kindly provide the complete questionnaire used here.

4. Kindly provide gender wise distribution for the respondents based on qualification to see if gender wise differences exist.

5. Exactly where was the study conducted is not clear, as the geographic variation may also need to be accounted.

6. Why no correlation statistics is performed?

**********

6. PLOS authors have the option to publish the peer review history of their article (what does this mean?). If published, this will include your full peer review and any attached files.

If you choose “no”, your identity will remain anonymous but your review may still be made public.

**Do you want your identity to be public for this peer review?**  For information about this choice, including consent withdrawal, please see our Privacy Policy.

Reviewer #1: No

Reviewer #2: **Yes:** Prof. Khalid Aboalshamat

Reviewer #3: No

**********

[NOTE: If reviewer comments were submitted as an attachment file, they will be attached to this email and accessible via the submission site. Please log into your account, locate the manuscript record, and check for the action link "View Attachments". If this link does not appear, there are no attachment files.]

While revising your submission, please upload your figure files to the Preflight Analysis and Conversion Engine (PACE) digital diagnostic tool, https://pacev2.apexcovantage.com/. PACE helps ensure that figures meet PLOS requirements. To use PACE, you must first register as a user. Registration is free. Then, login and navigate to the UPLOAD tab, where you will find detailed instructions on how to use the tool. If you encounter any issues or have any questions when using PACE, please email PLOS at figures@plos.org. Please note that Supporting Information files do not need this step.

Attachment Submitted filename: PLOS review.docx

# Author comment: Author response to Decision Letter 1

**DOI:** 10.1371/journal.pone.0350450.r002

1 Jul 2025

To

The Editor

Dear Prof,

Thank you for your valuable feedback; we confirm that our submission met the minimal data set requirements of PLOS One. The attached dataset (RawDATA.xlsx with the tab "Complete Raw Data") includes every individual-level data point that we used in the statistical analyses presented in the manuscript, specifically:

• Raw values used to calculate all of the reported means, standard deviations, frequencies, and percentages;

• The actual variables used to build tables and figures; in this case, demographic distributions and response frequencies;

• There was no data that came from images or point extraction from images.

We are attaching the raw data, in a supplemental file, with the full cleaned manuscript file. The dataset is completely annotated and corresponds directly with the variables in the manuscript, thus allowing full reproducibility of all results and conclusions.

Attachment Submitted filename: Comments Addressed.docx

# Peer review: Decision Letter 1

**DOI:** 10.1371/journal.pone.0350450.r003

**Authors:** Tanay Chaubal, Tanay Chaubal

12 Aug 2025

PONE-D-25-16205R1

Ocular Injury Awareness, Knowledge, and Safety Practices Among Dental Professionals, Students, and supporting Staff: A Cross-Sectional Analysis

PLOS ONE

Dear Dr. Mohmed Isaqali Karobari,

Thank you for submitting your manuscript to PLOS ONE. After careful consideration, we feel that it has merit but does not fully meet PLOS ONE’s publication criteria as it currently stands. Therefore, we invite you to submit a revised version of the manuscript that addresses the points raised during the review process.

==============================

**ACADEMIC EDITOR: I appreciate your efforts in responding to the reviewers' comments. However, there are few more queries raised by the reviewers' which need to be addressed.**

==============================

Please submit your revised manuscript by Sep 26 2025 11:59PM. If you will need more time than this to complete your revisions, please reply to this message or contact the journal office at plosone@plos.org. When you're ready to submit your revision, log on to https://www.editorialmanager.com/pone/ and select the 'Submissions Needing Revision' folder to locate your manuscript file.

Please include the following items when submitting your revised manuscript:

- A rebuttal letter that responds to each point raised by the academic editor and reviewer(s). You should upload this letter as a separate file labeled 'Response to Reviewers'.
- A marked-up copy of your manuscript that highlights changes made to the original version. You should upload this as a separate file labeled 'Revised Manuscript with Track Changes'.
- An unmarked version of your revised paper without tracked changes. You should upload this as a separate file labeled 'Manuscript'.

If you would like to make changes to your financial disclosure, please include your updated statement in your cover letter. Guidelines for resubmitting your figure files are available below the reviewer comments at the end of this letter.

If applicable, we recommend that you deposit your laboratory protocols in protocols.io to enhance the reproducibility of your results. Protocols.io assigns your protocol its own identifier (DOI) so that it can be cited independently in the future. For instructions see: https://journals.plos.org/plosone/s/submission-guidelines#loc-laboratory-protocols. Additionally, PLOS ONE offers an option for publishing peer-reviewed Lab Protocol articles, which describe protocols hosted on protocols.io. Read more information on sharing protocols at https://plos.org/protocols?utm_medium=editorial-email&utm_source=authorletters&utm_campaign=protocols.

We look forward to receiving your revised manuscript.

Kind regards,

Tanay Chaubal

Academic Editor

PLOS ONE

Journal Requirements:

If the reviewer comments include a recommendation to cite specific previously published works, please review and evaluate these publications to determine whether they are relevant and should be cited. There is no requirement to cite these works unless the editor has indicated otherwise.

[Note: HTML markup is below. Please do not edit.]

Reviewers' comments:

Reviewer's Responses to Questions

**Comments to the Author**

1. If the authors have adequately addressed your comments raised in a previous round of review and you feel that this manuscript is now acceptable for publication, you may indicate that here to bypass the “Comments to the Author” section, enter your conflict of interest statement in the “Confidential to Editor” section, and submit your "Accept" recommendation.

Reviewer #3: All comments have been addressed

Reviewer #4: (No Response)

**********

2. Is the manuscript technically sound, and do the data support the conclusions?

The manuscript must describe a technically sound piece of scientific research with data that supports the conclusions. Experiments must have been conducted rigorously, with appropriate controls, replication, and sample sizes. The conclusions must be drawn appropriately based on the data presented.

Reviewer #3: Yes

Reviewer #4: Yes

**********

3. Has the statistical analysis been performed appropriately and rigorously?

Reviewer #3: Yes

Reviewer #4: No

**********

4. Have the authors made all data underlying the findings in their manuscript fully available?

The PLOS Data policy requires authors to make all data underlying the findings described in their manuscript fully available without restriction, with rare exception (please refer to the Data Availability Statement in the manuscript PDF file). The data should be provided as part of the manuscript or its supporting information, or deposited to a public repository. For example, in addition to summary statistics, the data points behind means, medians and variance measures should be available. If there are restrictions on publicly sharing data—e.g. participant privacy or use of data from a third party—those must be specified.

Reviewer #3: Yes

Reviewer #4: Yes

**********

5. Is the manuscript presented in an intelligible fashion and written in standard English?

PLOS ONE does not copyedit accepted manuscripts, so the language in submitted articles must be clear, correct, and unambiguous. Any typographical or grammatical errors should be corrected at revision, so please note any specific errors here.

Reviewer #3: Yes

Reviewer #4: Yes

**********

6. Review Comments to the Author

Please use the space provided to explain your answers to the questions above. You may also include additional comments for the author, including concerns about dual publication, research ethics, or publication ethics. (Please upload your review as an attachment if it exceeds 20,000 characters)

Reviewer #3: Significant improvement in the article overall. the article has been modified nicely as per the reviewers comments

Reviewer #4: (No Response)

**********

7. PLOS authors have the option to publish the peer review history of their article (what does this mean?). If published, this will include your full peer review and any attached files.

If you choose “no”, your identity will remain anonymous but your review may still be made public.

**Do you want your identity to be public for this peer review?**  For information about this choice, including consent withdrawal, please see our Privacy Policy.

Reviewer #3: No

Reviewer #4: **Yes:** AMITHA BASHEER N

**********

[NOTE: If reviewer comments were submitted as an attachment file, they will be attached to this email and accessible via the submission site. Please log into your account, locate the manuscript record, and check for the action link "View Attachments". If this link does not appear, there are no attachment files.]

While revising your submission, please upload your figure files to the Preflight Analysis and Conversion Engine (PACE) digital diagnostic tool, https://pacev2.apexcovantage.com/. PACE helps ensure that figures meet PLOS requirements. To use PACE, you must first register as a user. Registration is free. Then, login and navigate to the UPLOAD tab, where you will find detailed instructions on how to use the tool. If you encounter any issues or have any questions when using PACE, please email PLOS at figures@plos.org. Please note that Supporting Information files do not need this step.

Attachment Submitted filename: Comments.pdf

# Author comment: Author response to Decision Letter 2

**DOI:** 10.1371/journal.pone.0350450.r004

21 Aug 2025

Response to Comments

We appreciate the Editor’s insightful and constructive comments, which have greatly enhanced our manuscript. Below, we offer a detailed response to each point, including references to specific sections of the revised manuscript where changes have been implemented.

Abstract

Comment 1.

The abstract is long and includes some repetition. Details about specific percentages could be shortened without losing clarity.

Response:

We have refined the abstract by eliminating redundant details and retaining only the essential percentages to emphasize key findings (e.g., awareness 86.1%, ocular injuries 62.5%, compliance 42.6%). Repetition has been removed, allowing the abstract to convey the core message more succinctly (see Abstract in the revised manuscript).

✅ Addressed.

Introduction

Comment 2.

The rationale for conducting this study in the Indian context is underdeveloped. There is little discussion of local adherence to protocols, institutional standards, or prior Indian studies.

Response:

We have revised the Introduction to strengthen the rationale within the Indian context. In particular, we highlight that the Dental Council of India does not mandate ocular safety training and cite several Indian studies (Uppal et al., Arvind et al., Nimma et al., along with regional surveys in Bengaluru and Maharashtra). These studies uncover systemic gaps, regional disparities, and insufficient adherence to protective practices across India (pages 3–5).

✅ Addressed.

Methodology

Comment 3.

Provide a clearer explanation of how potential sampling biases were mitigated.

Response:

We added explicit details on bias mitigation: initial survey distribution targeted both urban/rural and public/private institutions, state-level representation was monitored, and reminders were withheld once saturation was reached (see Sampling and Recruitment, page 8).

✅ Addressed.

Results

Comment 4.

The analysis is descriptive; no comparative testing between groups (e.g., gender, role).

Response:

We included comparative statistical analyses throughout the Results:

• Gender vs. role distribution (χ² = 34.7, p < 0.001, Table 1).

• Gender-based differences in ocular injury incidence, frequency, and causes (χ² tests with p-values, Table 3).

• Differences in patient safety practices across groups (χ² tests, Table 5).

These strengthen the analytical depth and go beyond descriptive reporting (pages 9–15).

✅ Addressed.

Discussion

Comment 5

The discussion is descriptive and lacks deeper reflection on why awareness doesn’t translate into practice. It also omits systemic, sociocultural, and policy barriers.

Response:

We substantially revised the Discussion to provide critical insights:

• Ergonomic and psychosocial barriers (e.g., PPE discomfort, fogging, communication issues, as noted by Malandkar et al.).

• Cultural influences (hierarchical modeling, appearance concerns, invulnerability bias).

• Systemic challenges (lack of Dental Council of India mandates, variability across institutions).

• Financial and misconception barriers (cost of visors, false sense of safety from spectacles).

• Policy implications (role of institutional audits, enforceable standards, affordability initiatives).

These revisions provide a comprehensive explanation of the knowledge–practice gap and highlight multi-tiered solutions (pages 16–20).

✅ Addressed.

Conclusion

Reviewer comment:

The conclusion does not outline specific future research directions or policy implications.

Response:

We revised the Conclusion to explicitly recommend:

• Longitudinal and interventional research (e.g., educational workshops, simulation-based interventions).

• Comparative regional studies to identify systemic barriers.

• Integration of ocular safety into undergraduate and postgraduate curricula as a core competency.

• Development of enforceable national standards, affordability initiatives, and institutional audits (page 21).

✅ Addressed.

References

Reviewer comment:

Ensure uniform formatting per PLOS ONE.

Response:

All references have been reformatted to ensure consistency with journal style. Numbering has been checked and aligned sequentially.

✅ Addressed.

Summary

All reviewer concerns have been comprehensively addressed in the revised manuscript through:

• Concise and focused abstract,

• Strengthened Indian context and justification,

• Clearer methodology with bias control,

• Comparative statistical analyses,

• Critical and multi-level discussion of barriers,

• Specific research and policy recommendations in conclusion,

• Standardized references.

We believe these revisions have significantly improved the manuscript and aligned it with the reviewer’s expectations.

Attachment Submitted filename: Response to Comments.docx

# Peer review: Decision Letter 2

**DOI:** 10.1371/journal.pone.0350450.r005

**Authors:** Tanay Chaubal, Tanay Chaubal, Tanay Chaubal

24 Mar 2026

<div>PONE-D-25-16205R2

Ocular Injury Awareness, Knowledge, and Safety Practices Among Dental Professionals, Students, and supporting Staff: A Cross-Sectional Analysis

PLOS One

Dear Dr.  Karobari,

Thank you for submitting your manuscript to PLOS ONE. After careful consideration, we feel that it has merit but does not fully meet PLOS ONE’s publication criteria as it currently stands. Therefore, we invite you to submit a revised version of the manuscript that addresses the points raised during the review process.

==============================

**ACADEMIC EDITOR:  I commend the authors on responding to the reviewers' comments. However, there are few more queries which need to be addressed.**

==============================

Please submit your revised manuscript by May 08 2026 11:59PM. If you will need more time than this to complete your revisions, please reply to this message or contact the journal office at plosone@plos.org. When you're ready to submit your revision, log on to https://www.editorialmanager.com/pone/ and select the 'Submissions Needing Revision' folder to locate your manuscript file.

Please include the following items when submitting your revised manuscript:

- A letter that responds to each point raised by the academic editor and reviewer(s). You should upload this letter as a separate file labeled 'Response to Reviewers'.
- A marked-up copy of your manuscript that highlights changes made to the original version. You should upload this as a separate file labeled 'Revised Manuscript with Track Changes'.
- An unmarked version of your revised paper without tracked changes. You should upload this as a separate file labeled 'Manuscript'.

-->If you would like to make changes to your financial disclosure, please include your updated statement in your cover letter. Guidelines for resubmitting your figure files are available below the reviewer comments at the end of this letter.

If applicable, we recommend that you deposit your laboratory protocols in protocols.io to enhance the reproducibility of your results. Protocols.io assigns your protocol its own identifier (DOI) so that it can be cited independently in the future. For instructions see: https://journals.plos.org/plosone/s/submission-guidelines#loc-laboratory-protocols. Additionally, PLOS ONE offers an option for publishing peer-reviewed Lab Protocol articles, which describe protocols hosted on protocols.io. Read more information on sharing protocols at https://plos.org/protocols?utm_medium=editorial-email&utm_source=authorletters&utm_campaign=protocols.

We look forward to receiving your revised manuscript.

Kind regards,

Tanay Chaubal

Academic Editor

PLOS One

Journal Requirements:

1. If the reviewer comments include a recommendation to cite specific previously published works, please review and evaluate these publications to determine whether they are relevant and should be cited. There is no requirement to cite these works unless the editor has indicated otherwise.

2. Please review your reference list to ensure that it is complete and correct. If you have cited papers that have been retracted, please include the rationale for doing so in the manuscript text, or remove these references and replace them with relevant current references. Any changes to the reference list should be mentioned in the rebuttal letter that accompanies your revised manuscript. If you need to cite a retracted article, indicate the article’s retracted status in the References list and also include a citation and full reference for the retraction notice.

Additional Editor Comments:

I commend the authors for their perseverance in responding to the reviewers' comments. However, there are few more queries which need to be addressed.

[Note: HTML markup is below. Please do not edit.]

Reviewer's Responses to Questions

**Comments to the Author**

1. If the authors have adequately addressed your comments raised in a previous round of review and you feel that this manuscript is now acceptable for publication, you may indicate that here to bypass the “Comments to the Author” section, enter your conflict of interest statement in the “Confidential to Editor” section, and submit your "Accept" recommendation.

Reviewer #5: (No Response)

**********

2. Is the manuscript technically sound, and do the data support the conclusions?

The manuscript must describe a technically sound piece of scientific research with data that supports the conclusions. Experiments must have been conducted rigorously, with appropriate controls, replication, and sample sizes. The conclusions must be drawn appropriately based on the data presented.

Reviewer #5: Yes

**********

3. Has the statistical analysis been performed appropriately and rigorously?

Reviewer #5: Yes

**********

4. Have the authors made all data underlying the findings in their manuscript fully available?

The PLOS Data policy requires authors to make all data underlying the findings described in their manuscript fully available without restriction, with rare exception (please refer to the Data Availability Statement in the manuscript PDF file). The data should be provided as part of the manuscript or its supporting information, or deposited to a public repository. For example, in addition to summary statistics, the data points behind means, medians and variance measures should be available. If there are restrictions on publicly sharing data—e.g. participant privacy or use of data from a third party—those must be specified.

Reviewer #5: Yes

**********

5. Is the manuscript presented in an intelligible fashion and written in standard English?

PLOS ONE does not copyedit accepted manuscripts, so the language in submitted articles must be clear, correct, and unambiguous. Any typographical or grammatical errors should be corrected at revision, so please note any specific errors here.

Reviewer #5: Yes

**********

6. Review Comments to the Author

Please use the space provided to explain your answers to the questions above. You may also include additional comments for the author, including concerns about dual publication, research ethics, or publication ethics. (Please upload your review as an attachment if it exceeds 20,000 characters)

Reviewer #5: The authors may consider briefly expanding the limitations section to explicitly acknowledge potential self-reporting and recall bias.

A short comparison with similar occupational safety studies in other healthcare professions could strengthen the discussion.

Clarification on whether institutional safety policies varied across participating sites would add useful context.

**********

7. PLOS authors have the option to publish the peer review history of their article (what does this mean?). If published, this will include your full peer review and any attached files.

If you choose “no”, your identity will remain anonymous but your review may still be made public.

**Do you want your identity to be public for this peer review?**  For information about this choice, including consent withdrawal, please see our Privacy Policy.

Reviewer #5: **Yes:** Akshay Ashok Katara

**********

[NOTE: If reviewer comments were submitted as an attachment file, they will be attached to this email and accessible via the submission site. Please log into your account, locate the manuscript record, and check for the action link "View Attachments". If this link does not appear, there are no attachment files.]

To ensure your figures meet our technical requirements, please review our figure guidelines: https://journals.plos.org/plosone/s/figures

You may also use PLOS’s free figure tool, NAAS, to help you prepare publication quality figures: https://journals.plos.org/plosone/s/figures#loc-tools-for-figure-preparation.

NAAS will assess whether your figures meet our technical requirements by comparing each figure against our figure specifications.

# Author comment: Author response to Decision Letter 3

**DOI:** 10.1371/journal.pone.0350450.r006

25 Mar 2026

Dear Prof,

We appreciate the Editors and Reviewers' insightful and constructive comments, which have greatly enhanced our manuscript. Below, we offer a detailed response to each point, including references to specific sections of the revised manuscript where changes have been implemented.

Reviewer 5 comments

Comment: The manuscript must describe a technically sound piece of scientific research with data that supports the conclusions. Experiments must have been conducted rigorously, with appropriate controls, replication, and sample sizes. The conclusions must be drawn appropriately based on the data presented.

Our Response: We thank the reviewer for this important comment. The manuscript has been revised to further clarify the study's methodological rigour. Specifically, we have detailed the questionnaire validation process, including pilot testing and internal consistency (Cronbach’s alpha = 0.81), and expanded the description of the a priori sample size calculation using OpenEpi to ensure adequate statistical power. We have also clarified the statistical approach, including the appropriate selection of tests based on data distribution and the use of post hoc corrections. Finally, the conclusions have been carefully aligned with the presented data, particularly the observed discrepancy between awareness, compliance, and incidence of ocular injuries, to ensure that all inferences are directly supported by the results.

Comment: The authors may consider briefly expanding the limitations section to explicitly acknowledge potential self-reporting and recall bias.

Our Response: We thank the reviewer for this valuable suggestion. The limitations section has been revised to explicitly acknowledge the potential for self-report bias, including socially desirable responses, as well as recall bias in participants’ reports of past ocular injuries and clinical practices. These additions improve the transparency and interpretability of the study findings.

Comment: A short comparison with similar occupational safety studies in other healthcare professions could strengthen the discussion.

Our Response: We thank the reviewer for this insightful suggestion. The Discussion section has been revised to include a brief comparison with occupational safety practices in other healthcare professions, particularly among physicians and nursing staff. This addition highlights that the observed knowledge–practice gap is consistent with broader trends in healthcare, thereby strengthening the contextual interpretation of our findings.

Comment: Clarification on whether institutional safety policies varied across participating sites would add useful context.

Our Response: We thank the reviewer for this important observation. The Discussion section has been revised to clarify that institutional safety policies were not directly assessed or stratified across participating sites. We have also acknowledged that variability in local protocols, enforcement, and resource availability likely exists and may have influenced the observed differences in compliance, thereby providing additional context to the findings.

Attachment Submitted filename: Response_to_comments_auresp_3.docx

# Peer review: Decision Letter 3

**DOI:** 10.1371/journal.pone.0350450.r007

**Authors:** Tanay Chaubal, Tanay Chaubal, Tanay Chaubal, Tanay Chaubal

22 Jun 2026

Ocular Injury Awareness, Knowledge, and Safety Practices Among Dental Professionals, Students, and supporting Staff: A Cross-Sectional Analysis

PONE-D-25-16205R3

Dear Dr. Mohmed Isaqali Karobari,

We’re pleased to inform you that your manuscript has been judged scientifically suitable for publication and will be formally accepted for publication once it meets all outstanding technical requirements.

Within one week, you’ll receive an e-mail detailing the required amendments. When these have been addressed, you’ll receive a formal acceptance letter and your manuscript will be scheduled for publication.

An invoice will be generated when your article is formally accepted. Please note, if your institution has a publishing partnership with PLOS and your article meets the relevant criteria, all or part of your publication costs will be covered. Please make sure your user information is up-to-date by logging into Editorial Manager at Editorial Manager® and clicking the ‘Update My Information' link at the top of the page. For questions related to billing, please contact billing support.

If your institution or institutions have a press office, please notify them about your upcoming paper to help maximize its impact. If they’ll be preparing press materials, please inform our press team as soon as possible -- no later than 48 hours after receiving the formal acceptance. Your manuscript will remain under strict press embargo until 2 pm Eastern Time on the date of publication. For more information, please contact onepress@plos.org.

Kind regards,

Tanay Chaubal

Academic Editor

PLOS One

Additional Editor Comments (optional):

Reviewers' comments:

Reviewer's Responses to Questions

**Comments to the Author**

1. If the authors have adequately addressed your comments raised in a previous round of review and you feel that this manuscript is now acceptable for publication, you may indicate that here to bypass the “Comments to the Author” section, enter your conflict of interest statement in the “Confidential to Editor” section, and submit your "Accept" recommendation.

Reviewer #5: All comments have been addressed

Reviewer #6: All comments have been addressed

**********

2. Is the manuscript technically sound, and do the data support the conclusions?

The manuscript must describe a technically sound piece of scientific research with data that supports the conclusions. Experiments must have been conducted rigorously, with appropriate controls, replication, and sample sizes. The conclusions must be drawn appropriately based on the data presented.

Reviewer #5: Yes

Reviewer #6: (No Response)

**********

3. Has the statistical analysis been performed appropriately and rigorously?

Reviewer #5: Yes

Reviewer #6: (No Response)

**********

4. Have the authors made all data underlying the findings in their manuscript fully available?

The PLOS Data policy requires authors to make all data underlying the findings described in their manuscript fully available without restriction, with rare exception (please refer to the Data Availability Statement in the manuscript PDF file). The data should be provided as part of the manuscript or its supporting information, or deposited to a public repository. For example, in addition to summary statistics, the data points behind means, medians and variance measures should be available. If there are restrictions on publicly sharing data—e.g. participant privacy or use of data from a third party—those must be specified.

Reviewer #5: Yes

Reviewer #6: (No Response)

**********

5. Is the manuscript presented in an intelligible fashion and written in standard English?

PLOS ONE does not copyedit accepted manuscripts, so the language in submitted articles must be clear, correct, and unambiguous. Any typographical or grammatical errors should be corrected at revision, so please note any specific errors here.

Reviewer #5: Yes

Reviewer #6: (No Response)

**********

6. Review Comments to the Author

Please use the space provided to explain your answers to the questions above. You may also include additional comments for the author, including concerns about dual publication, research ethics, or publication ethics. (Please upload your review as an attachment if it exceeds 20,000 characters)

Reviewer #5: This manuscript presents a timely and clinically relevant cross-sectional study evaluating ocular injury awareness, knowledge, and protective practices among dental professionals, students, and supporting staff. The topic is important because ocular hazards in dentistry remain underrecognized despite clear occupational risks from aerosols, splatter, chemicals, and projectiles. The study includes a substantial sample size and offers meaningful data regarding the gap between awareness and compliance.

Reviewer #6: (No Response)

**********

7. PLOS authors have the option to publish the peer review history of their article (what does this mean?). If published, this will include your full peer review and any attached files.

If you choose “no”, your identity will remain anonymous but your review may still be made public.

**Do you want your identity to be public for this peer review?**  For information about this choice, including consent withdrawal, please see our Privacy Policy.

Reviewer #5: **Yes:** Akshay Ashok Katara

Reviewer #6: No

**********

# Editor report: Acceptance letter

**DOI:** 10.1371/journal.pone.0350450.r008

**Authors:** Tanay Chaubal, Tanay Chaubal, Tanay Chaubal, Tanay Chaubal

PONE-D-25-16205R3

PLOS One

Dear Dr. Karobari,

I'm pleased to inform you that your manuscript has been deemed suitable for publication in PLOS One. Congratulations! Your manuscript is now being handed over to our production team.

At this stage, our production department will prepare your paper for publication. This includes ensuring the following:

* All references, tables, and figures are properly cited

* All relevant supporting information is included in the manuscript submission,

* There are no issues that prevent the paper from being properly typeset

You will receive further instructions from the production team, including instructions on how to review your proof when it is ready. Please keep in mind that we are working through a large volume of accepted articles, so please give us a few days to review your paper and let you know the next and final steps.

Lastly, if your institution or institutions have a press office, please let them know about your upcoming paper now to help maximize its impact. If they'll be preparing press materials, please inform our press team within the next 48 hours. Your manuscript will remain under strict press embargo until 2 pm Eastern Time on the date of publication. For more information, please contact onepress@plos.org.

You will receive an invoice from PLOS for your publication fee after your manuscript has reached the completed accept phase. If you receive an email requesting payment before acceptance or for any other service, this may be a phishing scheme. Learn how to identify phishing emails and protect your accounts at https://explore.plos.org/phishing.

If we can help with anything else, please email us at customercare@plos.org.

Thank you for submitting your work to PLOS ONE and supporting open access.

Kind regards,

PLOS ONE Editorial Office Staff

on behalf of

Dr. Tanay Chaubal

Academic Editor

PLOS One
